# The Effects of Lemon balm on Menstrual Bleeding and the Systemic Manifestation of Dysmenorrhea

**Published:** 2018

**Authors:** Parvaneh Mirabi, S. Hanieh Alamolhoda, Mansooreh Yazdkhasti, Faraz Mojab

**Affiliations:** a *Infertility and Reproductive Health Research Center, Health Research Institute, Babol University of Medical Sciences, Babol, Iran. *; b *Midwifery and reproductive health department, Nursing and midwifery School, Shahid Beheshti University of Medical Sciences, Tehran, Iran.*; c *Department of Midwifery, Faculty of Midwifery, Alborz University of Medical Sciences, Karaj, Iran.*; d *Pharmaceutical Sciences Research Center and School of Pharmacy, Shahid Beheshti University of Medical Sciences, Tehran, Iran.*

**Keywords:** Melissa officinalis, Bleeding, Menstrual cycles, Systemic manifestations of dysmenorrhea

## Abstract

We conducted a double-blind randomized placebo controlled trial to evaluate the impact of Lemon balm (*Melissa officinalis* L.) on the bleeding and systemic manifestations of menstruation. A total of 90 students were randomly assigned to treatment or placebo group. Bleeding and systemic manifestations were evaluated with a menstrual pictogram and multidimensional verbal scale before and during 2 consecutive menstrual cycles, respectively.

Statistical tests indicated that in both groups, the severity of the systemic symptoms associated with dysmenorrhea significantly decreased (*P* = 0.001). Among the systemic symptoms, the mean severity of fatigue, the lethargy, and nervous changes in the two groups decreased after the treatment, which was statistically significant. There was no significant difference between the two groups regarding fatigue in the three cycles, but there was a significant difference between the two groups regarding lethargy in the first cycle (*P* = 0.05) and the second cycle (*P* = 0.001) after the treatment. The present study demonstrated that *Melissa officinalis* decreases the severity of the systemic signs associated with menstruation. It showed that the herb does not increase the severity of bleeding and the duration of menstruation. However, it reduces the mean total score of the severity of all the systemic symptoms associated with dysmenorrhea.

## Introduction

Medicinal plants have long been used as healing substances in the treatment of conditions like menstrual bleeding everywhere across the world; Iran has been no exception. Dysmenorrhea, or painful menstruation, is a pathological pelvic complication and one of the most common problems in women (1, 2). Dysmenorrhea is one of the main factors that impair the quality of life and the social activities of young women. While it lasts, it decreases occupational and educational efficiency, especially when it is accompanied with symptoms such as headache, fatigue, nausea, vomiting, diarrhea, shivering, and muscular cramps. About 50% to 70% of women experience dysmenorrhea (3, 4). These uterine cramps are associated with one or more systemic symptoms in more than 50% of the cases, including nausea and vomiting (90%), fatigue (85%), diarrhea (60%), lower back pain (60%), and headache (45%). Dysmenorrhea is clearly associated with lower abdominal cramps, and even causes digestive problems and fatigue (5, 6).

The cause of primary dysmenorrhea can be attributed to the increased synthesis of prostaglandins, which is secreted from the uterine endometrium during menstruation. The causative agents of the cramp and the systemic symptoms include mental and psychological factors, endocrine factors, cervical factors, abnormal uterine activity, and also the overproduction and excessive secretion of prostaglandins. Since the endometrial concentrations of PGF2α and PGE2 are related to the severity of the gastrointestinal symptoms associated with dysmenorrhea, the theory of excess protein production and the secretion of prostaglandins are confirmed, and gain more credibility than the other causes (7, 8).

The prostaglandins work to contract the smooth muscles of many tissues, including the gastrointestinal system, the bronchi, and arteries; therefore, one of the symptoms associated with primary dysmenorrhea is digestive disorder, including nausea, vomiting, and diarrhea, which is suggested due to the spasm of the gastrointestinal muscles at the initiation of menstruation (3, 8, 9). The usual menstrual bleeding period is 4–6 days, but in most women, this period could be anywhere between two and eight days. The normal volume of menstrual bleeding is 30 milliliters. If it is more than 80 milliliters, it is unusual (10).

The Lemon balm* (Melissa officinalis*) herb, from Labiatae family, is a stable, slightly fluffy plant with a height of 30 to 80 cm, which usually grows beside the hedges on the edges of forests and on shady lands. They often grow in the wild. Animal models have been studied to ascertain the analgesic and sedative effects of this plant (11, 12). Some compounds were reported in lemon balm, such as volatile oil (0.02- 0.8%) (Chief components are citral and citronellal), glycosides, caffeic acid derivatives, flavonoids, terpene acids, etc (13).

In traditional medicine, *M.* *officinalis* has been described as a potent, sedative, anti-bacterial, antiviral, and intestinal antispasmodic, and is an effective medicine for treating migraine headaches, calming the intestinal muscles, improving fatigue due to menstrual disorders, calming the stomach contractions that cause vomiting, and calming nerve-induced stomachache, unilateral headaches, vertigo, vomiting during pregnancy, and bad temper in women (2, 14). Its hydroalcoholic extract has a sedative effect and antinociceptive effect in mice and stimulates and enhances sleep in the animals (12). The anti-inflammatory activity of this plant is achieved by rosemary acid and prevents the activity of C3 convertase (2, 15).

In addition to the softening and protection from spasms, the essential oil of this plant has antimicrobial and antifungal effects. Severe diarrhea and fever, sometimes accompanied by vomiting, are common symptoms of rotavirus in children. The use of this plant has been proposed to prevent the spread of the virus as an effective drug for the treatment of rotavirus gastroenteritis infections (15, 16). In spasms associated with diarrhea and colic, even in irritable bowel syndrome, lemon balm is used (17, 18).

According to Reiter *et al*., *M. officinalis *inhibited contractions in guinea pig ileum, rat duodenum and vas deferens, and rabbit jejunum. The essential oil also exhibited smooth muscle relaxant activity in tracheal muscle of guinea pig (19). In clinical trials with the extract of this plant, no adverse effects, unwanted effects, or allergies have been reported and its use is approved by the Food and Drug Administration (20, 21).

Apparently, due to its biochemical properties, *M. officinalis* can affect the systemic symptoms associated with dysmenorrhea. Regarding the prevalence and the mechanism of dysmenorrhea, and the significant digestive and neurological disorders, this study aimed at to study the effects of *M. officinalis* on menstrual bleeding and systemic symptoms of primary dysmenorrhea. The goal of this study is to address the need for an efficient, easy to use and inexpensive drug.

## Experimental

This study was a double-blind controlled trial. After approval from the Shahid Beheshti University of Medical Science Ethics Committee, this study was registered in IRCT with the code: IRCT201107096826N2.

All single female resident students in university, who had moderate to severe dysmenorrhea as deduced by a verbal multidimensional scoring system, were evaluated. The inclusion criteria were the following: single status, age between 18 and 26 years, having moderate to severe dysmenorrhea according to the McGill system, having symptoms associated with dysmenorrhea according to the verbal multidimensional scoring system, having no severe hemorrhage, having no known chronic disease, having no symptoms such as irritation, itching, and abnormal discharge, having regular menstrual cycles at intervals of 21–35 days, and having no history of pelvic inflammatory disease, myoma, and tumor.

The sample size was estimated to be 45 in group, based on similar studies (8), considering 95% confidence, 5% error, and 0.6 effect size, considering 10% loss from an initial sample size of 55 in each group in the follow-up phase. Initially, the research objectives and the protocol were explained to the students living in the dormitories. In case of willingness to participate in the study, written consent was obtained from them. The research tool used in this research for collecting data was a questionnaire, which was designed in two sections: 

The first section was completed in the first stage of the study and before the start of the treatment. The second section of the questionnaire, information forms 2 and 3, was related to the severity of bleeding and systemic symptoms, completed twice in two successive cycles by the research units. In order to determine the validity of the questionnaire and the information form, content validity was used and the reliability of the test was evaluated by the re-test method which resulted in (r = 0.8).

For the eligible participants, the demographics, including age, body mass index (BMI), exercise, the presence of stressor in the last six months, and the table of systemic symptoms and severity of bleeding were completed. 

The severity and the duration of bleeding were evaluated based on the Campbell and Munga menstrual pictogram (22) and the verbal multidimensional scoring system—scored from zero to 3—was used to evaluate the systemic symptoms.

The participants with a history of a specific disease, who needed to use a drug, had symptoms such as burning, itching, discharge, the presence of stressors in the last six months, irregular menstrual cycles, and mild dysmenorrhea were excluded from the study. The eligible individuals were divided equally into separate blocks (moderate and severe symptoms). In the next stage, the participants were matched in terms of the severity of the symptoms and randomly divided into two groups of drug and placebo.

*Randomization*

Computer-generated random numbers were used to allocate the participants to receive either *M. officinalis* or the placebo. Allocation sequence will be password-protected and only accessible to the one midwife not involved in the study. Capsules were prepared for both the groups in similar packages with the codes A and B and the questioner and participants were unaware of the drugs since only the codes A and B were detectable. The capsules were administered with the codes and the findings were documented in a separate form. The subjects and the researchers were uninformed of the groups.

The method of drug preparation: The *M. officinalis* samples were taken from the farms around Karaj (Alborz Province, Center of Iran) and after the identification and verification, in the School of Pharmacy, Shahid Beheshti University of Medical Sciences, they were ground by the electrical grinder. The resultant powder was extracted with ethanol 96% (maceration ×3), the extract was mixed with corn starch and then, the powdered extract was put into capsules (size 0) with a handy machine.

The placebo capsules containing cornstarch were made under the same conditions. For the *M. officinalis* group, the capsules containing 330 mg extract of the herb were given to the participants for three days from the beginning of menstruation, thrice daily over two cycles. The placebo group was given capsules containing corn starch with the same protocol. 

Each herbal capsule was standardized and contains 1.3 total flavonoids (rutin).

*Masking*

To achieve the purpose of blinding, the characteristics of the real drug and placebo should be identical in color, appearance and smell, so the capsules were similar in shape and package and we put capsules (lemon balm and placebo capsules) next to the each other for more contamination. 

In addition, envelopes containing the forms 2 and 3 were given to the participants, in order to determine the severity of the systemic symptoms, menstruation, severity, and the duration of bleeding. The forms were filled up over two cycles. The data regarding menstruation (menstrual blood loss, number of pads) and the systemic manifestation of dysmenorrhea were collected through these forms. The primary endpoints were the severity of the systemic symptoms and secondary endpoint was the severity of bleeding. 

Statistical analysis. Standard statistical procedures were carried out with the Statistical Package for Social Sciences (SPSS) version 21.0. The normality of the quantitative variables was revealed by the Kolmogorov-Smirnov test and the Student’s t-test was used to compare the quantitative variables. The outcomes were assessed in the ITT analyses.

The descriptive analysis was conducted for each variable including the frequencies, means, and standard deviations, and the percentage of the variables in samples. The Friedman statistical test was used to compare the bleeding and the systemic manifestation of dysmenorrhea between three cycles and the Mann Whitney test compared the findings between the two groups. Insignificant results of the Friedman test, the therapeutic cycles were compared in pairs by modification of α and the Wilcoxon test.

## Results

**Figure 1:**
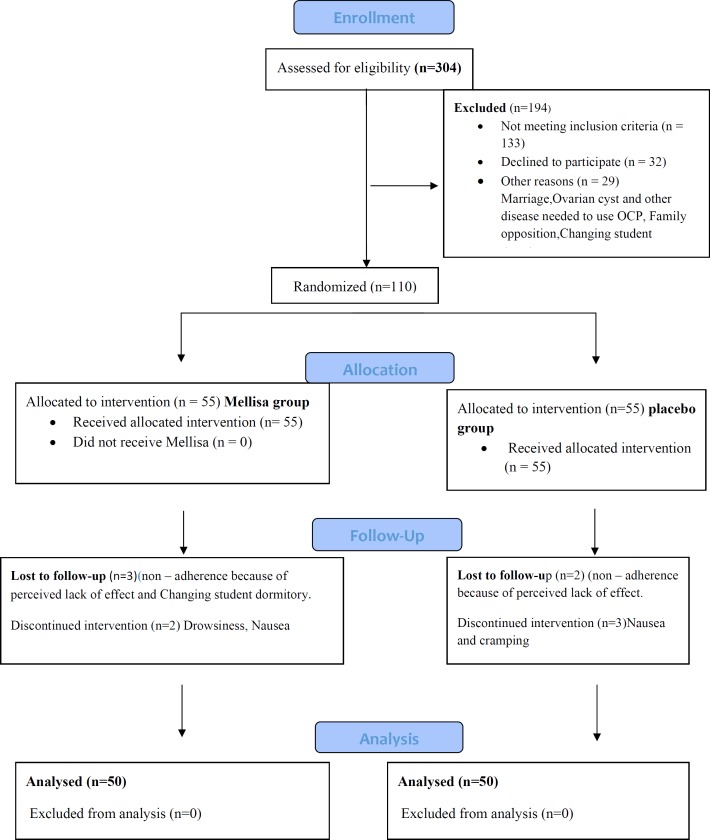
Flow of participants through the study

Of the 620 single female dormitory residents, 304 reported primary dysmenorrhea and of these, 119 students met the study’s eligibility criteria. Out of them, 110 agreed to participate. The final analysis included 100 students, 50 of whom received the *M. officinalis* and 50 received placebo (Figure 1, consort).

In Table 1 the demographic characteristics, body mass index, and the menstruation characteristics of the samples were presented, indicating that there was no significant difference between the two groups in terms of these variables and the two groups were homogeneous.

In both groups, the mean changes in the systemic symptoms associated with dysmenorrhea before and after the intervention were evaluated. The severity of the systemic symptoms associated with dysmenorrhea decreased significantly (*P* = 0.001).

Among the systemic symptoms, the mean severity of fatigue, the lack of energy and nervous changes in the two groups decreased after the treatment, which was statistically significant (Table 2). Then, the difference between the two groups of *M. officinalis* and placebo was evaluated before and after the first and second treatment cycles, by the Mann–Whitney test. There was no significant difference between the two groups regarding fatigue in the three cycles, but there was a significant difference between the two groups regarding lethargy in the first cycle (*P* = 0.05) and the second cycle (*P* = 0.001) after the treatment. The changes in the neurological variability were significantly different between the two groups only in the second cycle of the intervention (*P* = 0.01).

The mean severity of bleeding before and after the treatment was evaluated by the Friedman test, which revealed no significant differences (*P* = 0.52). 

To study the mean difference in menstrual periods between the two groups of drugs and placebo, before and after the first and second cycle after the treatment, the repeated measures test was used, which did not show a significant difference between the two groups in the three cycles (*P* = 0.27). Also, in order to determine and compare the duration of menstruation between the two groups, a t-test was used that showed no significant difference.

## Discussion

This experiment did not detect any evidence after the treatment on the severity of bleeding and the duration of menstruation; however the severity of the systemic symptoms associated with dysmenorrhea decreased significantly. Most women have a mean normal volume of menstrual bleeding, but if it is more than 80 milliliters, they could develop anemia. Given the prevalence of iron deficiency, anemia in Iranian girls and women is not so uncommon. People are very willing to receive herbal medicines and therefore, the physicians and midwives need to be fully aware of the side effects of herbal medicines in obstetrics and gynecology (23). Several papers have been published in this regard (24-26).

Given the traditional use of *M. officinalis* as a prescriptive drug and the fact that *M. officinalis* is a phytoestrogen, the essence of which can inhibit smooth muscle contractions (20), it is expected that *M. officinalis* increases the severity of menstrual bleeding, but the present study did not approve this theory.

These results are in agreement with Mir Ghafour *et al*., (2016), who showed that a treatment with *M. officinalis* did not decrease menstrual bleeding in students with premenstrual syndrome (27).

Based on our search, we did not find another study on this subject. But other medicinal plants such as valerian, cinnamon, and fennel, which are antispasmodic and have effects similar to *M. officinalis*—that has positive effects on dysmenorrhea and the systemic symptoms—did not affect the duration and severity of bleeding (8, 28, 29). Other important findings include the systematic signs. Herbal medicines reduce the level of prostaglandins, have a modulating effect on nitric oxide, increase the levels of beta-endorphin, block calcium channels, and improve circulation; thus, they are effective in the treatment of menstrual pain and the systematic manifestation of dysmenorrhea (4, 30).

*M. officinalis* is one of the oldest and most traditional herbal medicines. It is deemed to be antispasmodic, sedative/hypnotic, and it is used for strengthening the memory and for the relief of stress-induced headache (31, 32). The oil extracted from *M. officinalis* has an anti-inflammatory effect and issued for dysmenorrhea and its systematic sign (2, 33). In this study, with regard to the systemic symptoms associated with dysmenorrhea, the subjects in both groups had a similar severity of symptoms before the treatment and the severity of these symptoms changed after the treatment in both groups. The administration of *M. officinalis* reduced the severity of the systemic symptoms associated with primary dysmenorrhea, including fatigue, neurological changes, and lethargy. However, the severity of nausea and vomiting, diarrhea, headache, and fainting was not significantly different between the placebo and the treatment groups, although there was a decrease after the treatment. 

**Table 1:** Demographic characteristics of the participants

**Characteristics**	**Melissa**	**Placebo**	***P *** **value ** b
Age (year)	21.08±1.34	21.14±1.61	0.60
Menarche	13.30±1.35	13.46±1.05	0.50
Age of dysmenorrhea	15.62±2.23	15.66±1.84	0.92
Body mass index (kg/m2)^c^	21.73±3.07	22.59±3.82	0.24
Length of bleeding (day)	6.12±1.35	6±1.21	0.64
Length of Menstrual Cycle (day)	26.78±2.7	27.48±2.9	0.22

a Values are given as mean±SD unless otherwise indicated.

b t- test.

c Calculated as weight in kilograms divided by the square of height in meters.

**Table 2:** Severity of systemic signs associated with dysmenorrhea, as measured on a multidimensional verbal scale (score range 0–3)

**Systemic sign**		**Base line**	**st Cycle 1**	**nd Cycle 2**	***P*** **-value ** c
	Melissa	1.44±0.1	1.48±0.9	1.58±0.9	**0.02**
**Fatigue**	Placebo	1.50±1.07	1.46±1.03	1.80±0.8	0.61
	***P *** **-value ** b	0.33	0.97	0.77	**-**
	Melissa	0.62±1.01	0.50±0.81	0.50±0.76	0.82
**Nausea and vomiting**	Placebo	0.48±0.82	0.49±0.76	0.44±0.6	0.51
	***P *** **-value**	**0.05**	0.81	0.56	-
	Melissa	2.30±0.8	1.34±0.87	1.08±0.8	**>0.001**
**Lack of energy**	Placebo	2.14±0.9	1.66±0.7	1.60±0.84	**0.006**
	***P *** **-value**	0.35	**0.05**	**0.001**	**-**
	Melissa	0.66±1.02	0.64±1	0.72±1.01	0.69
**Headache**	Placebo	0.48±0.6	0.42±0.6	0.66±0.8	0.1
	***P *** **-value**	0.96	0.61	0.36	-
	Melissa	0.36±0.6	0.34±0.6	0.30±0.6	0.31
**Diarrhea**	Placebo	0.42±0.5	0.40±0.7	0.44±0.7	0.54
	***P *** **-value**	0.30	0.89	0.34	-
	Melissa	2.38±1.05	1.97±1.1	1.65±1.09	**>0.001**
**Mood swings**	Placebo	2.34±1.02	1.74±1.1	1.92±1.7	**>0.001**
	***P *** **-value**	0.64	094.	**0.01**	**-**
	Melissa	0.32±0.62	0.24±0.4	0.20±0.4	0.36
**Faint**	Placebo	0.38±0.7	0.34±0.6	0.30±0.6	**0.01**
	***P *** **-value**	0.89	0.14	**0.03**	**-**

a Values are given as mean±SD unless otherwise indicated

b Mann–Whitney U test

c Friedman test

**Figure 2:**
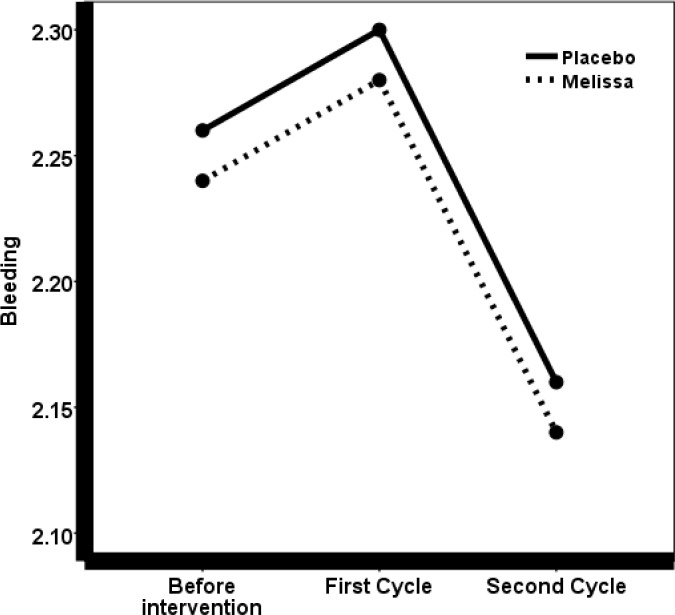
Comparison of the mean severity of menstrual bleeding previous and after intervention in two groups.

One of the symptoms was neurological changes. The treatment with *M. officinalis* and placebo both decreased the severity of the neurological changes, compared to before the treatment. But the decrease in the neurological changes was higher in the treatment group than in the placebo group, and in the second cycle after the treatment, there was a significant difference between the two groups. Therefore, according to these results, *M. officinalis* appears to mitigate the severity of neurological changes associated with dysmenorrhea.

Today, *M. officinalis* products are mainly used for mild forms of neurologic weakness, anxiety and stress, menstrual agitation, and other neurological changes, and most studies found to be one of its most important effects (12, **231**). Several reports have shown that *M. officinalis* can reduce the neurological symptom of premenstrual syndrome (PMS) through the GABA neurotransmitters. The GABA neurotransmitters have great inhibitory effects on the central nervous system and are essential for creating a balance between nervous stimulation and suppression of the brain’s normal function. It is reported that the brain’s GABA levels are highly associated with anxiety in such a way that benzodiazepines used as sedatives in the past decades imitate the GABA neurotransmitters. These medications result in sedative and anxiolytic effects by binding to the GABAergic receptors and changing other neurotransmitters of the brain, such as norepinephrine and serotonin (34-36). One study examined the effects of *M. officinalis* in the treatment of anxiety disorders. In this study, 20 men and women took 600 mg of a proprietary *M. officinalis* extract twice daily for 15 days. At the end of the study, 14 out of the 20 patients reported full remission of their anxiety (12).

In another study in 2009, the anti-depressant effects of *M. officinalis* were compared with imipramine and fluoxetine and in the end, the researchers concluded that *M. officinalis* has an antidepressant-like effect similar to imipramine and this may have a potential clinical value for the treatment of depression (37). In a study by Adefunmilayo *et al*., a significant decrease was observed in the severity of anxiety and neurological symptoms with the administration of *M. officinalis* (12).

Fatigue and lethargy are the other symptoms on which *M. officinalis* has an effect. In traditional books and some studies, the beneficial effects of this plant on fatigue and lack of energy have been expressed. The result of this study is, therefore, in line with the previous studies (36).

One of the systemic symptoms was a headache. In various studies and sources, *M. officinalis* has been introduced as an effective medication for headache and migraine (12, 31, 38). Fritz and Speroff also state that menstrual headaches are most often due to muscle contraction or psychological stress. Given that *M. officinalis* an anti-contraction and anti-stress herbal medicine and has been found to be effective in treating headache and migraine, it was expected that the herb would have a better effect in this regard. But the results of the present study were different from the previous findings (39). The mean severity of headache was lower in both groups after the treatment and although this decrease was not the same in the groups of *M. officinalis* and placebo, the difference was not statistically significant. It seems that *M. officinalis*, with this dose and three-day interval administration, does not improve the severity of headache associated with dysmenorrhea, but a higher dose or a greater number of administration days may have significant effect.

During menstruation, prostaglandin that contracts the smooth muscle of the uterus can cause symptoms of smooth muscle contraction else where in the body, including dyspnea due to bronchial constriction and diarrhea due to increased intestinal movements (39). Considering the effect of *M. officinalis* on calming the intestinal muscle (19), the digestive symptoms associated with dysmenorrhea including diarrhea,were expected to reduce. But in this study, the severity of diarrhea after the treatment did not change dramatically in both groups. In traditional medicine, this herb is effective in the treatment of diarrhea. According to our review, no study has been done in this regard. But studies have been conducted on plants similar to M. officinalis that have antispasmodic and sedative effects. One of the studies conducted on valerian did not have any effect on the gastrointestinal symptoms associated with dysmenorrhea (4). In the study by Jafari *et al*., the effects of valerian were studied on the reserpine rats and valerian was not effective in reucing diarrhea, nausea, and vomiting (40)

The main strength of this study is that it is the first time that *M. officinalis* has been assessed for its effectiveness on bleeding and the systemic manifestation of dysmenorrhea. Therefore, our results are innovative.

This study is subject to several limitations, including the fact that the information was self-reported by the participants and the responses of the people were reassured in this regard. The uncontrollable factors such as culture, genetic profile, and lifestyle, which influence the symptoms of dysmenorrhea, were the weak points of this study. Also, considering that this research was only conducted on the dormitory students with almost similar weather and nutritional conditions, it cannot indicate the state of all women of reproductive ages. The generalizability is compromised. 

## Conclusion

The present study demonstrated that *M. officinalis* decreases the severity of the systemic signs associated with menstruation, given that no adverse effects have been reported for *M. officinalis*. The present study also demonstrated that *M. officinalis* does not increase the severity of bleeding and the duration of menstruation. However, *M. officinalis*, without any special side effects, reduces the mean total score of the severity of all the systemic symptoms associated with dysmenorrhea. In the present study, the severity of the neurological symptoms, fatigue, and lethargy showed a significant decrease. Therefore, the herb can be administered safely for the management of the systemic manifestations of dysmenorrhea.
